# Reply to Pastore, E.P. Comment on “Korkmaz et al. A Deep Learning and Explainable AI-Based Approach for the Classification of Discomycetes Species. *Biology* 2025, *14*, 719”

**DOI:** 10.3390/biology15020107

**Published:** 2026-01-06

**Authors:** Aras Fahrettin Korkmaz, Fatih Ekinci, Şehmus Altaş, Eda Kumru, Mehmet Serdar Güzel, Ilgaz Akata

**Affiliations:** 1 Faculty of Health Sciences Nutrition, Dietetics Department, Şirinevler Campus, İstanbul Kültür University, Istanbul 34191, Türkiye; a.korkmaz@iku.edu.tr; 2 Institute of Artificial Intelligence, Ankara University, Ankara 06100, Türkiye; fatihekinci@ankara.edu.tr; 3 Department of Computer Engineering, Faculty of Engineering, Ankara University, Ankara 06830, Türkiye; 20291316@ogrenci.ankara.edu.tr (Ş.A.); mguzel@ankara.edu.tr (M.S.G.); 4 Graduate School of Natural and Applied Sciences, Ankara University, Ankara 06830, Türkiye; ekumru@ankara.edu.tr; 5 Department of Biology, Faculty of Science, Ankara University, Ankara 06100, Türkiye

The comment raises three principal themes: (i) safeguarding statistical independence through grouping at the level of specimen/collector/site prior to splitting, (ii) preventing selection bias via fully nested preprocessing and hyperparameter tuning, and (iii) assessing shortcut learning and reporting probability calibration for decision thresholding in ecological workflows [1]. The study addressed Discomycetes classification with a 2800-image corpus and a 60/20/20 train–validation–test protocol, and reported comparative CNN performance and XAI visualizations (Grad-CAM, Score-CAM) [2].

Attention to potential non-independence arising from near-duplicates, shared backgrounds, or repeated collection events is acknowledged [1]. The dataset was assembled to maximize heterogeneity across photographers, habitats, and illumination, and the fixed split (60%/20%/20%) was chosen to retain distributional diversity while isolating a held-out test set [2,3,4,5,6,7,8,9,10]. For future iterations, grouped partitioning will be prioritized to match the deployment setting:

- Specimen-grouped or event-grouped splitting (all images from a collection event withheld together);
- Site-blocked or photographer-blocked cross-validation to mitigate scene-level leakage;
- Geography-aware blocking (e.g., region or habitat strata) to better emulate out-of-domain transfer;
- Camera/EXIF-stratified folds to counter device-specific priors.

These designs align with hierarchical/spatial CV practices recommended for ecological data and directly address the dependency risks highlighted in the comment [1].

All transforms (normalization, resizing, augmentations) and optimizer schedules were confined to the training partition; validation was reserved for early stopping and hyperparameter selection; and the test set remained untouched for final reporting [1]. While this pipeline follows common deep learning practice in image recognition, a fully nested scheme will be adopted in expanded studies to provide more conservative generalization estimates: outer folds for evaluation, inner loops for fitting transforms and tuning, and frozen transforms applied to held-out folds before scoring [2]. This migration to nested CV will reduce selection bias and align the evaluation with rigorous protocols for structured ecological data.

The concern that high accuracy may arise from background or acquisition shortcuts rather than morphology is pertinent [1]. Class-activation analyses (Grad-CAM, Score-CAM) were used to interrogate focus regions for cap, hymenial surfaces, and other taxonomically meaningful traits [2,3,4,5,6,7,8]. To strengthen this line of evidence, subsequent work will include the following:

- Implement saliency sanity checks (model/label randomization tests) to verify explanation fidelity.
- Use perturbation-based localization and deletion/insertion metrics to quantify whether highlighted regions are causally important.
- Add counterfactual augmentations (background swaps, color-cast normalization) to stress-test reliance on contextual cues.
- Report probability calibration (temperature scaling/isotonic regression) with reliability diagrams and Expected Calibration Error (ECE), enabling threshold setting for biodiversity monitoring and curation pipelines [1].

These additions address the request for decisive tests beyond qualitative overlays and for calibrated outputs suitable for operational decision-making [1].

Generalization to new cameras, habitats, and independent collections is essential [1]. An expanded, multi-institutional repository (including herbarium-verified vouchers and geographically distinct field sets) is being curated to support the following:

- External validation on fully independent sources;
- Site-withheld evaluation where entire locales are unseen during training;
- Temporal holdouts (by season/year) to probe phenology-related drift;
- Domain-shift diagnostics (source-specific performance stratified by habitat, device, and photographer).

These experiments will complement the current fixed split and provide deployment-oriented evidence of robustness [2,3,4,5,6,7,8].

To aid re-use and auditability, forthcoming revisions will (i) enumerate transform parameters and their fit scope (training-only vs. applied) [2,3,4,5,6,7,8,9,10,11,12], (ii) list hyperparameter search spaces and selection criteria [2,3,4,5,6,7,8,9,10,11,12,13], (iii) publish per-class metrics (precision/recall/F1) and confusion matrices [2,3,4,5,6,7,8,9,10,11,12,13,14], and (iv) include calibration curves and threshold–utility analyses for practitioner selection in monitoring workflows [2,3,4,5,6,7,8]. Where permissible, code and trained weights will be shared to facilitate independent replication.

The present study established a comparative baseline across ten CNNs with XAI analyses on Discomycetes images [2]. Recognized limitations include (a) absence of fully nested CV, (b) lack of grouped/blocked splits, (c) qualitative emphasis in XAI without formal causality metrics, and (d) missing calibration reporting. The roadmap above targets each limitation with concrete methodological upgrades consistent with the comment’s recommendations [1].

The comment’s recommendations on grouped splitting, nested validation, explanation reliability, calibration, and external/site-withheld evaluation are well-taken and align with the next phase of this research program [1]. Within the current scope, processing was confined to training data, validation guided tuning, and a held-out test set was used for final reporting [2]. Future releases will incorporate grouped/nested protocols, quantitative XAI sanity checks, probability calibration, and independent validations to provide deployment-grade evidence for biodiversity and curation use cases.
